# Magnitude of infertility and associated factors among women attending selected public hospitals in Addis Ababa, Ethiopia: a cross-sectional study

**DOI:** 10.1186/s12905-022-01601-8

**Published:** 2022-01-11

**Authors:** Mekdes Akalewold, Getachew W. Yohannes, Ziyad Ahmed Abdo, Yonas Hailu, Aynye Negesse

**Affiliations:** 1 Department of Public Relation and Communication, Ethiopian Ministry of Health, Addis Ababa, Ethiopia; 2 Department of Public Health, Yekatit 12 Hospital and Medical Colleges, Addis Ababa, Ethiopia; 3 Department of Hygiene and Environmental Health, Ethiopian Ministry of Health, Addis Ababa, Ethiopia; 4 Department of Health Extension Program and Primary Health Care, Ethiopian Ministry of Health, Addis Ababa, Ethiopia

**Keywords:** Infertility, Magnitude, Associated factors, Addis Ababa, Ethiopia

## Abstract

**Introduction:**

The World Health Organization estimated that approximately 48 million couples and 186 million people are infertile worldwide. Although the problem of infertility is increasing worldwide, as well as in Ethiopia, there are limited studies done. Therefore, this study aims to determine the magnitude of infertility and the major risk factors in three governmental hospitals in Addis Ababa, Ethiopia.

**Method:**

An institutional-based cross-sectional study design was used to conduct the study. The participants were selected by using a systematic random sampling technique. Data were collected through an interview using a structured questionnaire. The data were entered into Epi Data version 3.1 and exported to SPSS version 25 for analysis. Logistic regression was used to identify the predictor variables. Statistical significance was considered at a *P* < 0.05 with an adjusted odds ratio calculated at 95% CI.

**Result:**

The overall prevalence of infertility was 27.6% (95%CI = 23.2, 32.0). Of these, 14.4% had primary infertility, and 13.2% had secondary infertility. Those whose duration of marriage was less than 60 months [AOR = 3.85; 95%CI 1.39, 10.64], had a history of fallopian tube obstructions [AOR = 8.27; 95%CI 2.36, 28.91], had irregular frequency of coitus [AOR = 37.4; 95%CI 11.29, 124.114], had more than one sex partner [AOR = 3.51; 95%CI 1.64, 7.54], had an abortion greater than 3 times [AOR = 6.89; 95%CI 1.28, 37.09], and had partners who currently consumed alcohol [AOR = 1.31; 95%CI 1.11, 1.86] were more likely to be infertile than their counterparts.

**Conclusion:**

According to the results of this study, the prevalence of infertility was high compared to the global estimate of the World Health Organization. The government, health care providers, and researchers should emphasize developing appropriate strategies, research, education, and awareness creation of infertility and its potential causes.

## Introduction

Infertility is a disease of the male or female reproductive system that is defined as the inability to conceive after 12 months or more of unprotected regular intercourse [1]. It is sometimes referred to as infecundity, sterility, or physiological infertility, which is defined by demographers as the inability of a man, woman, or couple to participate in reproduction [2]. The terms subfertility and infertility are often used interchangeably, but they are not the same [3]. Subfertility is defined as a condition in which a couple is less fertile than a normal couple. Pregnancy may take longer when a couple is subfertile. However, they can become pregnant on their own without medical help, as opposed to a condition called infertility [3, 4]. The source of infertility can be woman, man, both, or unexplained [5, 6]. Female infertility may be due to polycystic ovary syndrome, hormonal disorders, premature ovarian failure, genital infections, endometriosis, fallopian tube obstruction, congenital uterine anomalies, uterine synechiae, prolonged use of oral contraception, sociocultural factors, or other medical complications [7, 8]. Men are merely responsible for 20–30% of infertility cases but contribute to as many as 50% of the total cases [9].

Infertility is a concern, suffering, and stigma for couples facing this problem [10, 11]. It is more than a quality of life issue, with far-reaching consequences for public health, including psychological distress, social stigma, economic stress, marital disagreements, negative pregnancy outcomes, and later-onset adult diseases [12]. In addition to stigma and related emotional distress, studies in South Asia and the Middle East have demonstrated that infertility can be associated with increased interpersonal violence among infertile women [13, 14].

Due to lifestyle changes and the presence of various environmental pressures, the prevalence of infertility has increased significantly and has become the third most serious disease after cancer and cardiovascular diseases [15, 16]. WHO estimates estimate that between 48 million couples and 186 million people live with infertility globally [1]. It is estimated to affect between 8 and 12% of couples of reproductive age worldwide [17]. However, in some regions of the world, infertility rates are much higher, up to 30% in some populations [18]. This is especially true in many regions with a high incidence of infertility, such as South Asia, sub-Saharan Africa, the Middle East and North Africa, Central and Eastern Europe, and Central Asia [5, 17, 18].

A study found that the proportion of couples seeking medical attention is 56% in developed countries and 51% in developing countries [19]. Regardless of the widespread consequences of infertility, the provision of infertility medical care is limited in developing countries, including Africa, since greater attention is given to the problem of overpopulation and waiting to encourage childless couples to accept their condition [8].

In many African countries, the success of marriage overlies the ability of a woman to bear children, and being infertile leads to serious psychological trauma and social stigma [8]. Especially for women, infertility significantly reduces their quality of life, exposing more sexual partners, sexually transmitted diseases, increased sexual dysfunction, and bad relationships [20]. Although it is a common problem for both sexes, social blame and stigma are universally laid on women [21, 22]. In Ethiopia, marriage, parenthood, and children are highly valued, and women are defined in the context of motherhood, which limits their role in the private sphere [23]. A study performed in Butajira showed that the prevalence of primary and secondary infertility was 2.9% and 16.1%, respectively [24]. Another study performed in Ethiopia showed that the prevalence of primary infertility declined from 4.4% in 2000 to 3.3% in 2005, whereas secondary infertility increased from 4.3% in 2000 to 4.6% in 2005 [25]. A study performed in Addis Ababa regarding women’s infertile experience showed diverse negative emotional and psychological effects [26]. A study performed in Dessie identified that age at first pregnancy, age at menarche, menstruation flow in days, history of STI, and multiple sexual partners were the determinant factors of infertility [27].

Few studies have shown that the problem of infertility is increasing worldwide and in Ethiopia. To reduce the problem of infertility, individual and group efforts should be supported by evidence. As such, continuous studies should be done to generate strong evidence that supports tackling the problem. As part of future pieces of evidence, this study intends to assess the prevalence of infertility and its associated factors among women of reproductive age visiting selected health facilities in Addis Ababa.

## Methods and materials

### Study design and settings

An institution-based cross-sectional study design was used to conduct the study. Among the 12 public hospitals in Addis Ababa, the study was conducted in 3 of them. The selected hospitals were Black Lion Specialized Hospital, St. Paulos Specialized Hospital, Gandi Memorial General Hospital. Addis Ababa is the capital city of Ethiopia. According to the CSA 2019 projection, the city population was approximately 3,603,000. The male and female populations were 1,703,000 and 1,900,000, respectively [28]. Among females, 34.4% were women in the reproductive age group. The study was conducted from February 24 to March 24, 2021. Regarding infertility centers, all public hospitals except Amanuel specialized hospital in the city have obstetrics and gynecology wards that perform the first assessment, diagnosis, and some provision of treatments, but IVF is given nationally at St. Paulo’s hospital and a private clinic called Al-Hikma.

### Population and eligibility criteria

All women within the reproductive age group and attending the obstetrics and gynecology units either for infertility-related complaints or for obstetrical reasons in public hospitals in Addis Ababa were considered source populations. All reproductive-age women attending obstetrics and gynecology clinics in the selected hospitals were considered the study population. Married women who were in the reproductive age group (15–49 years) had gynecological problems and who tried to conceive for more than a year were included in the study. Women who were not in the reproductive age group, who had tried to conceive for less than one year, those using any form of family planning methods, and women with a history of hysterectomy were excluded from the study.

### Sample size and sampling strategy

The required sample size required for the study was calculated using a single population proportion formula with the following assumptions: 95% confidence level, 4% margin of error, and 21.2% proportion from a previous study performed in Ethiopia [29].

$$n = \frac{{(Z_{\alpha /2} )^{2} *p(1 - p)}}{{d^{2} }}$$

Accordingly, the calculated sample size was 401, and considering a 10% nonresponse rate, the total sample size was 441.

**Fig. 1:**
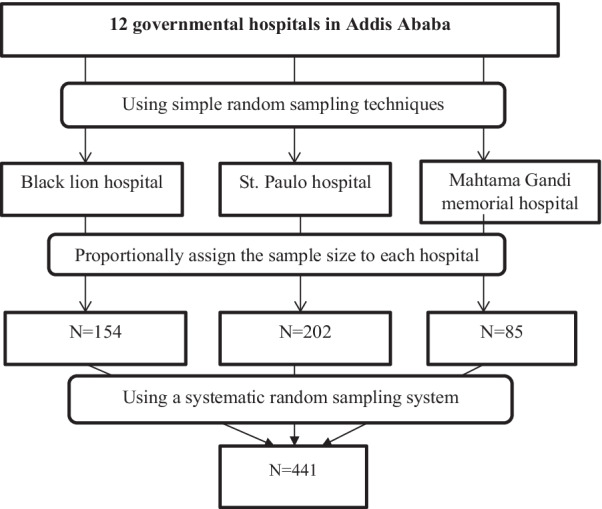
Schematic presentation of sampling procedure to select study participants

From a total of 12 government hospitals in Addis Ababa, three hospitals that have obstetrics and gynecology services were selected using simple random sampling techniques. Since these hospitals have a large flow of patients in their MCH unit, the sample size was allocated in proportion to the source population in each selected hospital using their six-month registered obstetrics and gynecology patient load. After estimating eligible female clients, a systematic random sampling technique was used to select participants from each hospital Fig. 1.

### Variables

- Dependent variable: Magnitude of infertility
- Independent variables: Socio demographic characteristics (age, sex, education level, occupation, economic status, duration of marriage, age at marriage), obstetrics and gynecological factors (parity, abortion, stillbirth, dysmenorrhea, irregular menses, operations, gynecologic disorders), sexual history **(**number of partners in the past, STI history, frequency of coitus**),** substance use, lifestyle and medical history **(**smoking, alcohol use, khat use, medical complications, stress**)**

### Operational definition

- *Infertility* is defined as if they fulfilled the following criteria.
  - Married for at least the past 12 months.
  - Either had not conceived in the past (primary infertility) or had conceived in the past but not in the last 12 months up to the date of the interview.
  - Who was not using contraceptives in the time one year before the date of the interview
  - Either breastfeeding or no breastfeeding but had been 12 months or more since she had seen her first post-delivery menses.
  - All the other women married for 12 months or more and who did not fulfil the above criteria were considered fertile.
- *Primary infertility* a condition in which a couple who has been married for at least one year and has never achieved conception despite having regular unprotected sexual intercourse
- *Secondary infertility* A condition in which a couple who had at least one previous conception irrespective of the outcome was trying to conceive for the last year or more had regular intercourse.
- *Regular coitus* Sexual intercourse at least two to three times per week.
- *Current substance use* Refer to the use of drugs or alcohol and including substances cigarettes, illegal drugs prescription drugs, inhalants, and solvents in the past 30 days.
- *Past substance use* Refer to the history of using drugs or alcohol, and include substances cigarettes, illegal drugs prescription drugs, inhalants, and solvents before one month.

### Data collection tools and quality control

The questionnaire was adapted from the reviewed previous literature [24, 30]. A structured questionnaire administered by an interviewer to eligible participants was used. The questionnaire was first prepared in English and then translated to Amharic, as the study subjects speak Amharic, and then back to English to check the consistency. Three female nurses and one health officer were recruited for data collection and supervision. To ensure data quality, adequate and appropriate training and guidance were provided to the data collectors and supervisors. The questionnaire was first pretested in 22 (5%) of the sampled population at Zewditu Hospital. After the pretest, the questionnaire was modified based on the findings related to clarity, wordings, logical sequence, skip patterns of the questions, and resources needed. During data collection, the supervisor checked the completeness and adequacy of the data collected daily and corrected them based on the problems identified. In addition, the investigator monitored and evaluated the overall quality of the data collection process.

### Data management and analysis

The data were entered into EPI data version 3.1 and then exported to SPSS version 23 for data management and analysis. Descriptive statistics of frequency, percentages, mean and other statistics were carried out. In addition, bivariate and multivariate analyses were used to identify significant variables. Variables with a *p* < 0.25 during bivariate analysis were included in multiple logistic regressions to identify the determinant factors of infertility. An odds ratio with 95% confidence intervals and a significance level of *p* < 0.05 were used to assess the association between determinant factors and the level of infertility. The output of the analysis is displayed by statistical tables and figures.

## Result

### Socio-demographic characteristics of women

A total of 409 respondents participated in the study, with a response rate of 93%. All women who participated in the study were married and had been living with their partners for 12 months or more. Most of the respondents (212, 51.8%) were in the age range of 30–39 years, and the mean age of women was 35 ± 6.14 years. Approximately 273 (66.7%) respondents had been living with their partners for more than 60 months. Approximately 73.3% of respondents were married at the age of 20 years and above. Approximately 123 (30.1%) respondents had primary education. The majority of women live in urban areas 326 (79.7%). Most women were housewives 192 (46.9%). Approximately 224 (54.8%) respondents’ average household income was 3000 birr or less (Table 1).

**Table 1:** Distribution of socio-demographic characteristics among women attending a gynecologic clinic in three public hospitals in Addis Ababa, Ethiopia, 2021

Variable	Possible options	Frequency	Percentage (%)
Women’s age	20–29	92	22.5
	30–39	212	51.8
	> 40	105	25.7
Duration of marriage	≥ 60 month	136	33.3
	> 60 month	273	66.7
Age at marriage	< 20 years	109	26.7
	≥ 20 years	300	73.3
Educational status	Illiterate	72	17.6
	Primary education	123	30.1
	Secondary education	102	24.9
	Diploma	51	12.5
	Degree and above	61	14.9
Residence	Urban	326	79.7
	Rural	83	20.3
Occupation	Trader	44	10.8
	Civil servant	83	20.3
	Private servant	71	17.4
	Housewife	192	46.9
	Self-employed	19	4.6
Working hour per day	Less than or equal to 8 h	172	77.1
	Greater than 8 h	51	22.9
Income (ETB)	≤ 3000	224	54.8
	3000–5000	93	22.7
	≥ 5000	92	22.5

### Obstetrics factors

Among the respondents, approximately 343 (83.9%) had been pregnant before. Of this, 162 (47.2%) had one to two children, while the rest had three or more children. On average, approximately 323 (94.2%) respondents were in the age group of 16 and above during their first pregnancy. More than 82.5% were pregnant before 12 months of initiating sexual intercourse. Of women who had been pregnant previously, 35 (10.2%) had faced stillbirths. Among the women who had a live birth, currently, only 24 (11.6%) women were breastfeeding, and only one-fourth had been breastfeeding for 12–24 months (Table 2).

**Table 2:** Obstetrics factors affecting infertility among women attending a gynecologic clinic in three public hospitals in Addis Ababa, Ethiopia, 2021

Variable	Possible options	frequency	Percentage (%)
History of pregnancy	Yes	343	83.9
	No	66	16.1
Parity(343)	No child	63	18.4
	1–2	162	47.2
	≥ 3	118	34.4
Age at first pregnancy (n = 343)	< 16 years	20	5.8
	≥ 16 years	323	94.2
Last pregnancy duration(n = 343)	< 12 month	60	17.5
	≥ 12 month	283	82.5
History of stillbirth(n = 343)	Yes	35	10.2
	No	308	89.8
Last pregnancy outcome(n = 343)	Abortion	120	35
	Stillbirth	21	6.1
	Live birth	202	58.9
Currently breastfeeding (n = 202)	Yes	24	11.6
	No	183	88.4
Duration of breastfeeding	< 6 month	2	8.3
	6–12 month	4	16.7
	12–24 month	18	75

### Gynecologic factors

Among respondents, 190 (46.5%) had a desire to become pregnant, and 124 (30.3%) of them had been trying to initiate pregnancy for the past 1 to 5 years. Approximately 181 (41.3%) had an irregular menstrual cycle, 131 (32%) had pain during menses (dysmenorrhea), and 112 (27.4%) had a previous operation in which the highest percentage (89, 80.2%) was related to either pelvic or abdominal problems. Of the respondents, 198 (48.4%) had been diagnosed with gynecological disorder. Approximately 85 (20.8%) respondents had either been evaluated or treated for infertility. Approximately 58.6% of women had an abortion before, out of which 114 (56.7%) had an abortion at least once in their lifetime (Table 3).

**Table 3:** Gynecological factors affecting infertility among women attending a gynecologic clinic in three public hospitals in Addis Ababa, Ethiopia, 2021

Variable	Possible options	frequency	Percentage (%)
Initiation of pregnancy(desire)	Yes	190	46.5
	No	219	53.5
Irregular menses	Yes	181	44.3
	No	228	55.7
Dysmenorrhea	Yes	131	32
	No	278	68
History of surgery	Yes	112	27.4
	No	297	72.6
Type of surgery(n = 112)	Pelvic or abdominal	89	80.2
	Ovarian or fallopian tube	18	16.2
	Others	4	3.6
Previous gynecological disorder	Yes	198	48.4
	No	211	51.6
Type of previous gynecological disorders (n = 198)	Uterine disorder	50	25.3
	Ovarian disorder	70	35.4
	PID	15	7.6
	Uterine cancer	22	11.1
	Other	41	20.7
Obstruction of fallopian tubes(n = 198)	Yes	34	17.1
	No	165	82.9
Endometriosis(n = 198)	Yes	9	4.5
	No	190	95.5
Previous infertility diagnosis	Yes	85	20.8
	No	324	79.2
Medication for ovary(n = 85)	Yes	45	52.9
	No	40	47.1
History of abortion(n = 343)	Yes	201	58.6
	No	142	41.4
Number of abortion(n = 201)	Once	114	56.7
	Twice	44	22
	Three times and more	43	21.39

### Contraceptive and sexual history

In the past 12 months before the data collection period, 26.7% of respondents were using different methods of contraception. Approximately 210 (51.3%) respondents had one partner, and 199 (48.7%) had more than one partner in the past. Among respondents, 82 (20%) had been treated for STIs before. Approximately 206 (50.4%) had sexual intercourse two to three times per week, while approximately 92 (22.5%) had sex only once per week (Table 4).

**Table 4:** Contraceptive and sexual history factors affecting infertility among women attending a gynecologic clinic in three public hospitals in Addis Ababa, Ethiopia, 2021

Variable	Possible options	frequency	Percentage (%)
Contraception use the past 12 month	Yes	109	26.7
	No	300	73.3
Number of partners in the past	One partner	210	51.3
	More than one partner	199	48.7
STI history	Yes	82	20
	No	327	80
Frequency of coitus	One time per week	92	22.5
	2–3 times per week	206	50.4
	More than 3 times per week	51	12.5
	One time in 2 weeks	14	3.4
	Rarely	29	7.1
	None	17	4.2

### Substance use, lifestyle, and medical history

Among respondents, approximately 23 (5.6%) and 13 (3.2%) were past and current alcohol users, respectively. Approximately 0.2% and 0.5% reported a history of smoking cigarettes and current Khat chewing, respectively. Approximately 51 (22.9%) respondents spent more than 8 h at work except for housewives. Approximately 29 (7.1%) and 22 (5.4%) had a history of thyroid disorder and diabetes, respectively (Table 5).

**Table 5:** Substance use, lifestyle, and medical history among women attending a gynecologic clinic in three public hospitals in Addis Ababa, Ethiopia, 2021

Variable	Possible options	frequency	Percentage (%)
Alcohol past users	Yes	23	5.6
	No	386	94.4
Alcohol current users	Yes	13	3.2
	No	396	96.8
Cigarette past users	Yes	1	0.2
	No	408	99.8
Cigarette current users	Yes	1	0.2
	No	408	99.8
Khat current users	Yes	2	0.5
	No	407	99.5
Thyroid disorder	Yes	29	7.1
	No	380	92.9
Diabetes	Yes	22	5.4
	No	387	94.6

### Partner’s substance use history

Among respondents’ partners, approximately 72 (17.6%) and 25 (6.1%) were past alcohol and cigarette users, respectively. Approximately 79 (19.3%) and 21 (5.1%) were current alcohol and cigarette users, respectively. Sixty-four (15.6%) of the women’s partners currently use Khat (Table 6).

**Table 6:** Partner’s substance use history affecting infertility among women attending a gynecologic clinic in three public hospitals in Addis Ababa, Ethiopia, 2021

Variable	Possible options	frequency	Percentage (%)
Alcohol past users	Yes	72	17.6
	No	337	82.4
Alcohol current users	Yes	79	19.3
	No	330	80.7
Cigarette past users	Yes	25	6.1
	No	384	93.9
Cigarette current users	Yes	21	5.1
	No	388	94.9
Khat current users	yes	64	15.6
	no	345	84.4

### Partner’s medical and surgical history

Among respondents partners, 45 (11%) face difficulty during intercourse. Approximately 28 (6.8%) partners currently experience chronic hypertension or abnormal cholesterol. Approximately 35 (8.5%) of the respondent’s partners had other major medical illnesses, such as diabetes. Approximately 16 (3.9%) of their partners had operations in the past, of which 9 (56.3%) had appendectomy surgery (Table 7).

**Table 7:** Partner’s medical and surgical history affecting infertility among women attending a gynecologic clinic in three public hospitals in Addis Ababa, Ethiopia, 2021

Variable	Possible options	frequency	Percentage (%)
Erectile dysfunction	Yes	45	11
	No	364	89
Current hypertension or abnormal cholesterol	Yes	28	6.8
	No	381	93.2
Specific chronic disease	Hypertension	26	89.7
	Abnormal cholesterol	3	10.3
Other major diseases	Yes	35	8.6
	No	374	91.4
Specific major illness	Diabetes	19	54.3
	Kidney disease	4	11.4
	HIV	7	20
	Asthma	3	8.6
	Cardiovascular disease	2	5.7
Past surgical history	Yes	16	3.9
	No	393	96.1
Type of surgery	Appendectomy	9	56.3
	Testicular	2	12.5
	Abdominal	3	18.8
	Cardiac	2	12.5

### Magnitude of infertility

**Fig. 2:**
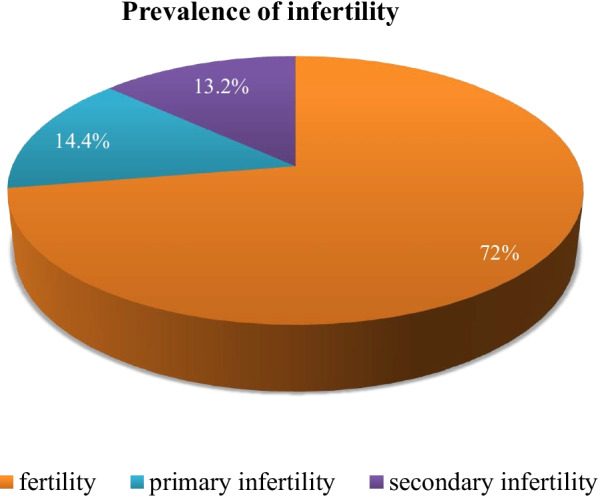
Magnitude of infertility among women attending gynecologic clinics in three public hospitals in Addis Ababa, Ethiopia, 2021

Among respondents, 113 (27.6%) (95% CI = 23.2, 32.0) were identified as infertile. Hence, 59 (14.4%) and 54 (13.2%) had primary and secondary infertility, respectively (Fig. 2).

### Factors associated with infertility

To identify the factors associated with infertility among women attending gynecologic clinics, first, bivariate regression analysis was performed. At this level, variables with a *p* value < 0.25 were included for multiple logistic regression analyses. Accordingly, the results of multiple regression analysis showed that covariates such as duration of marriage, history of fallopian tube obstruction, frequency of coitus, number of sex partners, number of abortion times, and partner’s current history of alcohol consumption were significantly associated with infertility in women (Table 8).

**Table 8:** Factors associated with infertility among women attending a gynecologic clinic in three public hospitals in Addis Ababa, Ethiopia, 2021

Variables	Infertility		COR (95% CI)	AOR (95% CI)
	Fertile N (%)	Infertile N (%)		
*Duration of marriage*				
≤ 60 month	91(66.9)	45(33.1)	1	1
> 60 month	205(75.1)	68(24.9)	0.671(0.428,1.052)	3.848(1.392,10.640)**
*History of fallopian tube obstruction*				
Yes	9(26.5)	25(73.5)	9.059(4.077,20.131)**	8.269(2.365,28.909)^*^
No	287(76.5)	88(23.5)	1	1
*Frequency of coitus*				
Regular	179(88.2)	24(11.8)	1	1
Irregular	117(56.8)	89(43.2)	5.673(3.416,9.424)**	37.4(11.298,124.114)**
*Number of sex partners*				
1	161(76.7)	49(23.3)	1	1
≥ 1	135(67.8)	64(32.2)	1.558(1.006,2.411)*	3.512(1.636,7.538)**
*History of STI*				
Yes	53(64.6)	29(35.4)	1.583(0.945,2.652)	0.912(0.269,3.093)
No	243(74.3)	84(25.7)	1	1
*Number of abortion*				
Once	93(81.6)	21(18.4)	1	1
Twice	42(95.5)	2(4.5)	0.454(0.146,1.410)	0.612(0.272,1.375)
Three times and more	39(90.7)	4(9.3)	2.154(0.373,12.425)	6.886(1.279,37.086)*
*Partner’s current alcohol history*				
Yes	64(81.0)	15(19.0)	1.555(1.302,2.021)	1.315(1.116,1.858)*
No	232(70.3)	98(29.7)	1	1

CI, confidence interval; COR, crude odds ratio; AOR, adjusted odds ratio

^*^P value < 0.05; **p value < 0.01

## Discussion

Infertility is a common medical problem that affects 5–8% of couples in developed countries and 5.8% to 44.2% in developing nations [31, 32]. According to this study, the magnitude of infertility among women attending gynecologic clinics in three public hospitals in Addis Ababa, Ethiopia, was 27.6%. Of these, 14.5% and 13.2% face primary and secondary infertility, respectively. The finding of this study is higher than that of a study performed in China in which the prevalence of infertility was 22.1% [33]. It is also higher than a similar study done in Cameroon in which the prevalence of infertility was 19.2% [34], also higher than a study done in Burkina Faso[35]. However, the finding of this study is much lower than the finding of a study done in Central Africa with a prevalence of 61% secondary infertility. Similarly, the finding is lower than that of a study performed in Arer city, Saud Arabia, in which the total prevalence of infertility among women attending the outpatient and inpatient departments in Maternity and Children’s Hospital was 65.3%; of these women, 19.8% had primary infertility and 80.2% had secondary infertility [22]. The observed difference may be due to variance in a different data collection and measurement tool, use of different cut-off points, the difference in sample size, sampling methods used, and variation in study participants.

Various studies have shown that too long a marriage period and old age in the couple can reduce their chances of having a new child [7]. The findings of this study show that the odds of infertility among the reproductive age group of women who attended obstetrics and gynecology units and whose duration of marriage was > 60 months were greater than those whose duration of marriage was ≤ 60 months. These have a great connection with the age of women. This means that women who stay in marriage for a long time have the probability of interring or close to menopausal age.

Fallopian tubes are two thin tubes, one on each side of the uterus, that help to transport a fertilized egg from the ovaries to the uterus. When an obstruction prevents the egg from traveling down the tube, women will have a blocked fallopian tube, also known as tubal factor infertility[33]. This can occur on one or both sides and is the cause of infertility in up to 30% of infertile women [36]. The results of this study also support this reality. Compared to those who had no history of fallopian tube obstruction, those who had a history of fallopian tube obstruction were more infertile. This study is supported by other studies that identified fallopian tube problems as a cause of infertility [8, 22].

Sexual factors referred to as sexual dysfunction of each couple included veganism, the method and time of sexual intercourse, and any sexual problem that prevents pregnancy [21]. Infertility is defined as the inability to conceive after 12 months of regular unprotected sexual intercourse [11]. In support of this reality, the results of this study show that the odds of infertility among those who had irregular coitus were greater than those among those who had regular coitus. In support of this finding, a study in China shows that couples aiming to conceive through regular unprotected sexual intercourse have an 85–90% chance of achieving pregnancy within 1 year, reaching > 90% in 2 years [37].

Having multiple sexual partners is not particularly unusual for most young people in the world today, and it has not been reported as an unhealthy practice [7]. However, these have a strong association with infertility. The findings of this study show that those who had more than one sexual partner were more likely to be infertile than those who had only one sexual partner. Similarly, a study performed in Dessei, Ethiopia, showed that women who had multiple sexual partners had a 5.3 times greater chance of infertility than those who did not have multiple sexual partners [23].

Abortion can bring wide public health and socioeconomic problems [38, 39]. Induced abortion may be safe or unsafe abortion. Worldwide, approximately 22 million unsafe abortions occur yearly. From this, 98% is occurring in developing countries [40]. Different studies also show that abortion can affect the fertility status of women. The results of this study show that the number of abortions affects the fertility level of women. As such, those who had abortions greater than or equal to 3 times were 6.89 times more likely to be infertile than those who had only one abortion.

Consuming excessive alcohol affects fertility by reducing testosterone levels, follicle-stimulating and luteinizing hormone, and raising estrogen levels, which reduce sperm production, shrinking the testes, which can cause impotence or infertility. Changing gonadotropin release affects sperm production [41, 42]. Comparable to this scientific evidence, the results of this study show that those who had a partner who currently does not consume alcohol and those who had a partner that currently consumes alcohol were more likely to be infertile.

## Conclusion

According to this study, the prevalence of infertility, both primary and secondary, was high compared to the WHO worldwide estimate. Infertility was significantly associated with duration of marriage, fallopian tube obstruction, frequency of coitus, number of sex partners, number of abortions, and partner’s past alcohol history. Unless emphasis is given, infertility issues will result in more public health problems. Women and their husbands should pay great attention to the possible factors that will lead to infertility. The government, health care providers, and researchers emphasize developing appropriate strategies, research, education, and awareness creation about infertility, its possible causes, and prevention methods.

## Data Availability

The datasets used and/or analyzed during the current study are not publicly available but are available from the corresponding author on reasonable request.

## Abbreviations

AOR: Adjusted odds ratio
CI: Confidence interval
CSA: Central Statistical Agency
HIV: Human Immunodeficiency Virus
IVF: In Vitro Fertilization
MCH: Maternal and child health
PID: Pelvic inflammatory disease
REC: Research and Ethical Committee
SPSS: Statistical Package for the Social Sciences
STI: Sexually transmitted infection
WHO: World Health Organization
